# Assessment of Coumarin Levels in Ground Cinnamon Available in the Czech Retail Market

**DOI:** 10.1100/2012/263851

**Published:** 2012-06-18

**Authors:** Jana Blahová, Zdeňka Svobodová

**Affiliations:** Department of Veterinary Public Health and Toxicology, Faculty of Veterinary Hygiene and Ecology, University of Veterinary and Pharmaceutical Sciences Brno, Palackého 1-3, 612 42 Brno, Czech Republic

## Abstract

The objective of this study was to determine the coumarin content of ground cinnamon purchased from retail markets in the Czech Republic. No sample was labelled with information on the botanical source, but, in some cases, the countries of origin were specified. For comparison, a single cinnamon sample imported directly from a plantation in Sri Lanka that came from *Cinnamomum verum* was analyzed. Results from 60 ground cinnamon samples comprising twelve brands confirmed a high content of coumarin, with mean levels ranging from 2 650 to 7 017 mg *·* kg^−1^. The high coumarin content confirmed that these cinnamon samples obtained from cassia cinnamon were in contrast to the sample from Sri Lanka, which was coumarin-free.

## 1. Introduction

Cinnamon is among the world's oldest known spices with many uses in the food processing, pharmaceutical, and cosmetics industries. It is produced from the dried inner bark of several tropical evergreen trees of the genus *Cinnamomum*. In the USA and European markets, two varieties of cinnamon, Ceylon and cassia, are available [1, 2].

Ceylon cinnamon, also known as “true cinnamon,” is obtained from *Cinnamomum verum* J. S. Presl. (syn. *Cinnamomum zeylanicum* Nees) indigenous to Sri Lanka and southern India. Cassia cinnamon has several origins, the important ones being Chinese cassia (*Cinnamomum cassia *Blume, syn. *Cinnamomum aromaticum *Nees) cultivated mainly in southern China, Burma, and Vietnam, and Indonesian cassia (*Cinnamomum burmanini* Blume), mainly from Indonesia and Sumatra [2–4]. The quality and price of cinnamon depend on factors such as botanical source, climatic conditions, and methods of harvesting and production. True cinnamon has a characteristic flavour different from cassia, being slightly sweeter and not as strong. Another important difference is in the coumarin content which may have a negative effect on human health and occurs in high content in cassia cinnamon. Cassia cinnamon contains up to 1% coumarin, whereas true cinnamon contains only a trace, about 0.004% [1–3]. Due to the high cost of true cinnamon, cassia cinnamon is often used in the food industry, although in some countries, substitution of cassia for true cinnamon is prohibited. It is therefore necessary to use analytical methods to discriminate between the two main types and to detect food adulteration.

Coumarin (benzo-*α*-pyrone) is a naturally occurring substance found in a wide variety of plants with pleasant flavour (tonka bean, vanilla, woodruff, etc.) including citrus fruits or green tea, but the main source of coumarin in the human diet is cinnamon [1, 5]. Synthetic coumarin was formerly widely used as a food additive, but the practice was prohibited by the US Food and Drug Agency in 1954, based on reports of hepatotoxicity to laboratory animals. The European Commission specified a maximum level of 2 mg·kg^−1^ of coumarin in food and beverages, with the exception of special caramels, alcoholic beverages, and chewing gum (10, 10, and 50 mg·kg^−1^, resp.) [6]. This Council Directive was replaced in 2008 by Regulation EC 1334/2008, defining higher maximum limits for cinnamon-containing food (desserts, traditional, and/or seasonal baked goods, and breakfast cereals including muesli) [7]. The direct addition of coumarin to food and beverages is prohibited, and any contribution must be through the use of natural flavourings. No limits have been established specifically for cinnamon.

Although adverse effect in humans (e.g., mild dizziness, diarrhoea, vomiting) after coumarin exposure is rare and generally associated with high doses administered in clinical therapies, recent studies report a connection of coumarin with liver tumours in rats and mice and with Clara cell toxicity and lung tumours in mice [8]. Coumarin was at first suspected to have genotoxic and carcinogenic effects in humans, but new toxicological data have shown it to be nongenotoxic and made it possible to define a tolerable daily intake [9]. It is believed that most humans possess a major pathway for the metabolism of coumarin (the 7-hydroxycoumarin pathway) that differs from that in rats (the 3,4-coumarin epoxidation pathway) in which a reactive epoxide is formed. The 3,4-coumarin epoxide route has been linked to the hepatotoxicity and carcinogenic effects of coumarin observed in long-term studies in rats and mice [10, 11]. In 2004, the European Food Safety Authority (EFSA) recommended a coumarin daily intake limit of 0–0.1 mg·kg^−1^ body weight per day.

The aim of this study was to assess the botanical source, true or cassia cinnamon, and the coumarin content in ground cinnamon available in the Czech retail market. 

## 2. Materials and Methods

### 2.1. Materials and Instrumentation

Coumarin (≥99%, HPLC), ammonium acetate (≥99%), methanol, and acetonitrile were obtained from Sigma-Aldrich (Sigma-Aldrich, Prague, Czech Republic). All used solvents were HPLC purity grade. Deionized water was prepared daily using Aqua Osmotic purification system (Aqua Osmotic, Tišnov, Czech Republic). Liquid chromatography analysis was performed using the HPLC system Alliance 2695 (Waters, Milford, MA, USA) and a diode array detector 2996 (Waters). 

### 2.2. Cinnamon Samples

Sixty ground cinnamon samples (twelve brands in five packages—J. C. Horn, Cimet, Avokádo, Fenik, Nadir, Spice Cellar, Mikado, Euroshoper, Vitana, Orient, Mammita, and Sindibád) were purchased from supermarkets and a specialized spice market. All five packages of one brand were purchased from the same dealer at the same time. None was labelled to indicate whether the contents were true or cassia cinnamon. In five cases, the country of origin was specified (generally Indonesia, India, or Vietnam) (Table 1). As a control and for comparison, we analysed a sample derived from *Cinnamomum verum* imported directly from a plantation in Sri Lanka. Analysis of this sample demonstrated that true cinnamon contains a low level of coumarin, most often below the limits of detection.

### 2.3. Sample Extraction

A 1.0 g sample of ground cinnamon was added to a 50 mL volumetric flask, filled with extraction solvent (methanol : water, 4 : 1), and stirred for 30 min at room temperature. After extraction, the sample aliquot was filtered through a disposable syringe filter (nylon, 0.45 *μ*m) and directly injected into the HPLC system. Each sample was prepared in duplicate.

### 2.4. Chromatography

The method for analysing the amount of coumarin in ground cinnamon samples was developed by Sproll et al. [9]. A Waters HPLC system with UV detector (279 nm) and Empower software was used. Separation was performed on a Kinetex 2.6 *μ*m, C18, 10 nm, 150 × 4.6 mm (Phenomenex, Torrance, CA, USA) with a mobile phase consisting of 5 mmoL·L^−1^ ammonium acetate buffer and acetonitrile/methanol (1 : 2). The elution mode was in an isocratic program with a flow of 0.6 mL·min^−1^. Under these conditions, the retention time of coumarin was 3.6 min. The injection volume was 5 *μ*L, and the column temperature was 35°C. 

### 2.5. Validation Parameters

The quantification of coumarin was performed by external calibration. Standard stock solution of coumarin (1 000 mg·L^−1^) was prepared in methanol/water (1 : 1) and stored at 4°C. Nine working standard solutions were prepared in methanol/water (1 : 1). All of the working standard solutions were injected onto the HPLC five times, and the average areas were used to generate the standard calibration curve. The calibration curve linearity was evaluated between 0.5 and 200 mg·L^−1^. Linear regression analysis of the external calibration curve was carried out by chromatographic Empower software (Waters), and the equation used to calculate the amount of coumarin in cinnamon samples was *y* = 30 315*x* + 13 944 (*R* ^2^ = 0.9996). The limit of detection (3*σ*) was 0.1 mg·L^−1^. Analysis repeatability was assessed from ten measurements of the same cinnamon samples by the same analyst under the same analytical conditions. The relative standard deviation of this measurement was 0.93%. Injection repeatability was studied from ten replicate injections of the same standard solution; the relative standard deviation was less than 0.4%. To study the reliability and suitability of used method, recovery experiment was carried out. To the cinnamon sample solution (50 mL), 1 mL of the standard solution, which contained 500 mg·L^−1^, was added. The recovery of method was 96%. 

### 2.6. Statistical Analysis

Statistical program Unistat 5.1 software (Unistat, London, United Kingdom) was used for calculation of basic statistical parameters (mean, standard deviation, relative standard deviation, minimum and maximum).

## 3. Results and Discussion

The coumarin content in samples is reported in Table 1. As expected, coumarin was detected in all samples. The mean content of 5 samples of each brand varied in the range 2 650 to 7 017 mg·kg^−1^. The coumarin content indicated that all samples obtained in the Czech retail markets were from cassia cinnamon. The coumarin content of a sample imported directly from Sri Lanka was below the limit of detection, confirming its origin as *Cinnamomum verum*. 

Although all packages of one brand were purchased in the same supermarket or specialized spice market at the same time, in case of samples 1 and 9, high variability among packages (RSD = 8.45 and 17.71%, resp.) was observed. In the sample with the highest variability, the coumarin content of these five samples ranged from 2 600 to 3 902 mg·kg^−1^. In other cases, the relative standard deviations were below 3%. One of the possible explanations is an inhomogeneity of ground cinnamon in manufactory. Woehrlin et al. [12] confirmed a large variation in coumarin levels especially in cassia bark samples from the same package as well as in trees imported directly from Indonesia. In cinnamon stick analyses, these researchers found coumarin levels to vary considerably within the same package, ranging from 300 to 9 000 mg·kg^−1^ (package A) and from 130 to 10 900 mg·kg^−1^ (package B). The bark from the same segment of a single tree imported directly from Indonesia may contain levels of coumarin ranging from undetectable to high [12]. Because commercially available cinnamon powder is produced by grinding bark derived from several trees, a smaller variation of coumarin content in the powder samples was found.

The levels of coumarin observed in the cinnamon samples are similar to those obtained in other studies. Sproll et al. [9] assessed the coumarin level in cinnamon and in cinnamon flavoured products (mulled wine, milk-containing food, bakery products, and breakfast cereals) in Germany. They reported that the majority of ground cinnamon available in the retail trade is cassia cinnamon and cinnamon without specification of origin, with coumarin content ranging from 2 880 to 4 820 and 0 to 8 790 mg·kg^−1^, respectively. They also analyzed five samples of true cinnamon which were coumarin-free. In the prepared foods investigated, appreciable amounts of coumarin were only found in cereals and bakery products. The highest coumarin content was found in cinnamon star cookies, a popular Christmas cookie. Eighty-five percent of all cookies sampled contained more than 2 mg·kg^−1^. The mean content was 25 mg·kg^−1^, and a maximum content of 88 mg·kg^−1^ was detected. Lungarini et al. [13] reported that about 70% of the tested ground cinnamon samples marketed in Italy originated from cassia cinnamon, whereas 85% of whole bark cinnamon samples were derived from true cinnamon.

In the Czech Republic, coumarin is monitored in cinnamon and in cinnamon-containing food and the results published annually in the Bulletin of the Ministry of Agriculture. In 2008 and 2009, twenty samples of ground cinnamon were analyzed from retail markets and all the samples were positive for coumarin with levels ranging from 1 170 to 5 719 mg·kg^−1^. Enhanced content of coumarin was found in some bakery products and breakfast cereals. The highest content was found in short pastry with a content of 60.5 mg·kg^−1^ [14, 15].

He et al. [16] dealt with authentication and quantitative analysis on the chemical profile of cassia bark. They confirmed that the determination of coumarin, cinnamaldehyde, cinnamic acid, and cinnamyl alcohol may be used for rapid reliable authentication and quality assessment of cassia bark.

In summary, all ground cinnamon samples purchased in the Czech retail markets were positive for coumarin with a mean content of 3 856 mg·kg^−1^. Although no sample was labelled as cassia cinnamon, it was evident that all of the samples came from cassia. It was confirmed that cinnamon available in the Czech retail market is predominantly cassia cinnamon, while true cinnamon was obtained only in specialty shops. Because cassia cinnamon contains high content of coumarin, heavy consumption of this spice may result in doses exceeding the tolerable daily intake. The greatest concern appears to be the coumarin in bakery products. Sproll et al. [9] reported that children could reach the tolerable daily intake by consuming 3-4 cinnamon star cookies (reported coumarin level of 88 mg·kg^−1^) of a typical weight 5 g, while an adult would need to eat approximately 10 cookies to reach the upper limit.

## Figures and Tables

**Table 1:** Basic statistical parameters of coumarin content in samples of each ground cinnamon purchase from Czech retail markets (SD: standard deviation, RSD: relative standard deviation).

Sample number	Source location	Mean [mg·kg^−1^]	SD	RSD [%]	Min.	Max.
1	Indonesia	3 477	294	8.45	2 952	3 624
2	Indonesia	3 141	8	0.26	3 132	3 151
3	Unknown	5 188	52	1.00	5 098	5 222
4	Unknown	5 796	56	0.97	5 703	5 848
5	Unknown	7 017	52	0.74	6 960	7 057
6	Indonesia/India	3 519	44	1.25	3 463	3 572
7	Indonesia	3 194	62	1.94	3 113	3 278
8	Unknown	3 108	33	1.06	3 069	3 140
9	Unknown	2 975	527	17.71	2 600	3 902
10	Unknown	2 937	63	2.14	2 834	2 987
11	Indonesia/Vietnam	2 650	78	2.94	2 571	2 734
12	Unknown	3 243	28	0.86	3 214	3 283
